# Turning over New Leaves

**Published:** 1888-10-27

**Authors:** 


### Turning Over New Leaves.

[Books for Review should be sent to The Editor, The Lodge, Porchester Square, W.]

**Medicine and Surgery at Guy's Hospital.***

In the short space allotted to a review in a hebdomadal journal it is impossible to do justice to such annual reports as those emanating from Guy's Hospital. In the exordium to a paper on two undescribed dislocations at p. 117 of the Report, Mr. Clement Lucas remarks: "The pages of the Guy's Hospital Reports of former years are so laden with the original observations of acute observers, that one who has a claim to be there represented must esteem it a high privilege. . . . No place indeed seems better suited for the publication of cases which are intended to take a permanent position in the history of the profession." To the first of these observations we give unqualified assent, but as regards the second the expression of a doubt may be perhaps pardoned. For Guy's Hospital Reports, notwithstanding a goodly list of upwards of four hundred subscribers, cannot be said to reach the masses of the profession. Yet it would be well if the Reports were read, marked, and inwardly digested by every medical man, not only in the three divisions of the British isles, but also wherever British medicine and surgery has penetrated. The cost, with postage, is only six shillings in Great Britain, and seven shillings abroad, and the type is worthy of the material—no inconsiderable inducement to the jaded practitioner, who is so frequently obliged to look into professional literature after an arduous day's work.

In Memoriam.

The present volume opens with two articles *in memoriam* of old Guy's men—Dr. Walter Moxon, and Mr. Cooper Foster—articles kindly written, and a fitting tribute to the worth of two members of the profession, who each, in his own special line, has left a mark on British medicine and surgery.

Foreknowledge of the Course of a Disease.

Dr. Pye Smith next gives his observations on "Prognosis; or, The Foreknowledge of the Future Course or Event of a Disease." There are some maladies, which, if unrelieved, will sooner or later terminate fatally; such, for example, as stone in the bladder, aneurism of the aorta, and almost as certainly ovarian tumour, and strangulated hernia; although, indeed, we have once known strangulated hernia to terminate in sloughing, and recovery of the person. There are also diseases, as, for instance, cataract and some kind of tumours, which, although not terminating fatally, will not disappear under the *vis medicatrix naturæ*. But in most other maladies, before a prognosis can be rightly given, the constitution and diathesis of the patient must be attentively considered. The prognosis of diabetes, of pneumonia, and of typhus is greatly determined by individual peculiarities. Age is one of the conditions on which much depends. Certain specific maladies are most dangerous in the aged, and least so in childhood and youth. On the other hand, there are maladies in which the reverse obtains. Habits, again, must often affect prognosis, and drunken habits, especially so in Bright's disease, in pneumonia, and in surgical injury. Turning from the patient to the disease, the severity of febrile symptoms holds the first place as affecting prognosis. Of special symptoms we have convulsions and hæmorrhagic rashes, both of serious evil import; but the labial herpes which appears concomitant with various maladies is generally regarded as a favourable sign. Writing of gout Dr. Pye Smith says, gout "does not fly to the stomach," an assertion which will be satisfactory to gouty subjects; but, on the other hand, we are told that hereditary gout is seldom thrown off, especially in the case of women, a remark which will not be quite so satisfactory to sufferers from this prevalent complaint. The prognosis of diseases of the nervous system is confessedly very difficult, yet Dr. Pye Smith gives some valuable general rules, and some few definite grounds for particular cases. As regards diseases of the lungs, it is pointed out that "from the unequivocal testimony of the dead-house," phthisis is a curable malady. The prognosis of diabetes is now generally admitted to depend almost entirely on age; in some few old persons it appears scarcely to shorten life. The same is more or less correct regarding cancer. To one aphorism of Dr. Pye Smith we venture deferentially to take exception. He tells us diarrhœa is dangerous only in infants and in persons above sixty. This may be correct in temperate climates, but tropical diarrhœa cannot be so spoken of.

The next article is by Dr. George Savage, "On some Modes of Treatment of Insanity as a Functional Disorder." Dr. Savage believes in the existence of a large number of cases of insanity which rather deserve to be considered as depending on functional disorder than on disease of the brain or nervous system, and he is strongly of opinion that, as function makes the organ, so disordered function may destroy the organ. His treatment of such cases is to endeavour to stimulate the mental functions from without, on the same principle that massage and galvanism are employed to restore muscular function.

"On Dental Surgery in General Hospitals" is a paper by Mr. Newland Pedley, and we fully agree with the author that lack of opportunity produces a condition of ignorance on matters dental among medical men. Mr. Pedley advocates dental surgery being represented on its highest lines in general hospitals. It may be doubted, however, if medical students have time to devote to mechanical dentistry, although undoubtedly they should know more of dentistry generally than they now do.

Dr. L. E. Shaw has a paper on "Cases of Hæmorrhage Occurring During Treatment of Acute Rheumatism by Salicylate of Soda." The toxic effect of this remedy has always been regarded as inconvenient, although not prohibitory of its use. Headache, deafness, tinnitus, vomiting, epistaxis, rashes, and various cardiac symptoms have frequently been noticed under the use of salicylate. Now Dr. Shaw records retinal hæmorrhage and hæmaturia. But in 32 per cent. of cases in which the drug has been used none of these unpleasant symptoms presented. In the cases in question twenty-grain doses were given. It is quite possible that the patients possessed a peculiar idiosyncrasy, or antipathy to the drug, and therefore would be affected by small doses. This is the case with other drugs, quinine for example. There are persons who cannot take a grain of quinine without suffering from sore throat, nettle rash, and other unpleasant effects, while in larger doses it excites exactly the symptoms mentioned above as brought on by salicylate. Iodide of potassium and ipecacuanha are other medicines to which peculiar antipathy sometimes exists.

Reminiscences of Provincial Surgery.

The next article, bearing this heading, is by a veteran "Old Guy's Man," Mr. Crompton of Birmingham. Mr. Crompton details various operations performed under circumstances which might only be expected to be present in uncivilised countries—not even on the modern battlefield, where, as a rule, better assistance would be forthcoming than Mr. Crompton seems to have obtained in his capacity as a provincial operating surgeon. Mr. Crompton also tells us of operations performed before the days of chloroform, and details several cases where the patient himself held the light for the surgeon, looking on complacently while the knife and saw did their work. "Is this courage or insensibility to pain?" asks Mr. Crompton, to which perhaps the correct reply would be, a little of both. Mr. Crompton does not, however, tell us only of his successes. He has the courage to record his failures also. He operated for umbilical hernia, which a post-mortem disclosed to be gallstone. On this case Mr. Crompton comments: "Though I was wrong as to my diagnosis, I was justified in my operation. I suppose a surgeon of the present day would say he must make another exploratory incision, and passing his hand into the abdomen he would probably have found the gallstone. . . . And the old lady of seventy-five would have died just the same." Of cancer Mr. Crompton remarks that, a great proportion of cases, whether operated on or not, end in about three or four years. The experience, therefore, of the veteran Birmingham surgeon is scarcely in accord with the dictum of at least one German surgeon whose name has been recently prominently before the public, and who considers cancer curable.

Next, Mr. L. A. Dunn contributes an interesting and well-related case of fractured pelvis, which occasioned extravasation of urine and perinæal fistula.

Diabetes Mellitus.

One of the most elaborate articles in the series is "Diabetes Mellitus," by Dr. H. J. Campbell. It is almost a complete monograph on the subject, but space will not admit of more than a glance at some few interesting points. Dr. Campbell

* Edited by N. Davies-Colley, M.A., M.C., and W. Hale White, M.D. Vol XLIV., being Vol. XXIX. of the third series. 1888. (London: J. and H. Churchill.)

mentions that diabetes was described by the ancient Eastern author Susruta, and he also states that the malady is still "strikingly common" in Ceylon and in some parts of India, although it occurs chiefly in temperate zones. This, however, may be doubted. Both Chevers and Moore, in their recently-published works on Indian diseases, show how very common it is in India. Chevers states that nearly every family of the better classes in Bengal has lost one or more of its members from diabetes. Again, as regards heredity, Dr. Campbell observes that, on looking through the records of Guy's Hospital, it is surprising to find how frequently the parents have been quite healthy. But the authors named above insist on the hereditary nature of the disease. As regards causes, wet, cold, and chill, in the opinion of patients, hold the first place. But violent exercise, too much saccharine food, alcohol, and syphilis must be credited with exciting causation. In the treatment of diabetes nearly every drug has been used. Even giving large quantities of sugar, on what principle we know not, has been tried. But it is questionable if many kinds of treatment really do much good. Dieting is not by any means always successful, and diabetic foods are not always what they are professed to be. Dr. Campbell does not tell us anything about saccharin for diabetes, which is an omission probably due to the article being written previous to the introduction of saccharin. The fact is, amelioration may take place under various treatments, but cure seldom. But diabetes in the aged, as mentioned in a previous part of this review, is less dangerous than when it occurs to young persons.

Deformities—Fractures.

Mr. Arbuthnot Lane next discourses on the "Causation, pathology, and physiology of several of the deformities which develop during young life," such as knock-knee, flat-foot, curvature of the spine, &c. This very able and illustrated paper is followed by "Cases of Angina Pectoris," by Dr. Goodhart, and by "Cocaine in Dental Surgery," by Mr. Pedley. There is next a case of "Friedereich's Disease," by Dr. Newton Pitt. The terminal articles form a lengthy illustrated account by Messrs. Lane, Poland, and Dunn, of "Abnormalities observed in the dissecting-room of Guy's Hospital during the seasons '85-86, and '86-87;" and on "Compound Fracture of Long Bones," by Mr. Davies-Colley. In the wards of Guy's Hospital, during a period of six years, Mr. Davies-Colley treated sixty-one compound fractures, the death-rate being less than 3 per cent. It is remarked that in few departments of surgery has greater improvement been effected during the last twenty years than in the treatment of compound fracture. The tabular statements accompanying this article, demonstrate the large amount of surgical practice coming under the care of only one of the surgeons of Guy's, and amply justify its publication in an enduring form. While admitting that good results may be obtained by several methods of treatment, Mr. Davies-Colley has seen no reason to deviate from the practice originally introduced by Sir Joseph Lister, and he has endeavoured to carry out the principle of Lister as completely as he could. But Mr. Davies-Colley generously attributes the good results to the great care which his dressers have taken in carrying out the treatment.

In concluding this brief commentary on No. XLIV. of Guy's Hospital Reports, we reiterate that there is a store of information on the different subjects treated, evidencing that the mine of practical knowledge afforded by the hospital is now worked most efficiently—as efficiently even as the forefathers of the profession could desire. Those who, as Mr. Clement Lucas observes, in former years enriched Guy's Hospital reports with original observations, excavated from the same, although a more shallow mine, than the modern one.

**Foster's Physiology.***

When a book has reached a fifth edition, it requires little commendation to the public. It has evidently found its audience, and Professor Foster's "Text-book of Physiology" is known and approved by most students of that science. Though less copious than Stirling's work, it excels it in lucidity of style, and is therefore better adapted to the requirements of those who are just entering on the study of physiology. The illustrations are admirably clear, and as simple as the nature of the subject permits, and the book, as a whole, now that it is revised up to date, will prove an admirable companion to the student for supplementing the lectures he hears in the class-room.

* A Text book of Physiology. By M Foster, M.A., M.D., LL.D, F.R.S., Pr fessor of Physiology in the University of Cambridge, and Fell w of Trinity College, Cambridge.

